# Hallmarks of Cancer Expression in Oral Lichen Planus: A Scoping Review of Systematic Reviews and Meta-Analyses

**DOI:** 10.3390/ijms232113099

**Published:** 2022-10-28

**Authors:** Miguel Ángel González-Moles, Carmen Keim-del Pino, Pablo Ramos-García

**Affiliations:** 1 School of Dentistry, University of Granada, 18071 Granada, Spain; 2 Instituto de Investigación Biosanitaria ibs.GRANADA, 18012 Granada, Spain

**Keywords:** oral lichen planus, oral cancer, malignant transformation, hallmarks of cancer, molecular, biomarkers, scoping review, systematic review, meta-analysis

## Abstract

Oral lichen planus (OLP) is a common chronic inflammatory disease of unknown etiology and likely autoimmune nature that is currently considered an oral potentially malignant disorder, implying that patients suffering from this process are at risk of developing oral cancer in their lifetime. The molecular alterations that develop in OLP and that make the affected oral epithelium predisposed to malignancy are unknown, although, as in other autoimmune diseases (ulcerative colitis, primary biliary cirrhosis, etc.), they may be linked to oncogenesis-promoting effects mediated by the inflammatory infiltrate. So far there is no in-depth knowledge on how these hallmarks of cancer are established in the cells of the oral epithelium affected by OLP. In this scoping review of systematic reviews and meta-analyses the state of evidence based knowledge in this field is presented, to point out gaps of evidence and to indicate future lines of research. MEDLINE, Embase, Cochrane Library and Dare were searched for secondary-level studies published before October 2022. The results identified 20 systematic reviews and meta-analyses critically appraising the hallmarks tumor-promoting inflammation (*n* = 17, 85%), sustaining proliferative signaling (*n* = 2, 10%), and evading growth suppressors (*n* = 1, 5%). No evidence was found for the other hallmarks of cancer in OLP. In conclusion, OLP malignization hypothetically derives from the aggressions of the inflammatory infiltrate and a particular type of epithelial response based on increased epithelial proliferation, evasion of growth-suppressive signals and lack of apoptosis. Future evidence-based research is required to support this hypothesis.

## 1. Introduction

In 2000, Hanahan and Weinberg [1] proposed to the scientific community a series of characteristics that would be key to distinguish neoplastic cells, such that the characterization of a cell as such would rest on the expression of these oncogenic signatures. The particularity of these distinctive characteristics lies in the fact that their appearance is neither globally nor drastically acquired by a cell but is achieved during a multi-step process, from the initial stages of cancer, even in premalignant stages, to the development of a fully established tumor. Today, it is known that the expansion and clonal selection of cell lines is the main mechanism of the progressive acquisition of the hallmarks of cancer and that the increase in cellular proliferative activity and the genomic instability that this entails are decisive in this process [1]. The final proposal [2] includes six hallmark features (maintaining proliferative signaling, evading growth suppressors, resisting cell death, allowing replicative immortality, inducing angiogenesis, activating invasion and metastasis), two enabling features (genome instability and mutation, and tumor-promoting inflammation) and two emerging features (deregulating cellular energy and immune system bypassing). The main consequence of the hallmarks of cancer research is that it has helped us to better understand the biopathogenic mechanisms involved in the initiation and progression of cancer and precancerous lesions, which is also applicable to oral carcinogenesis.

**Figure 1:**
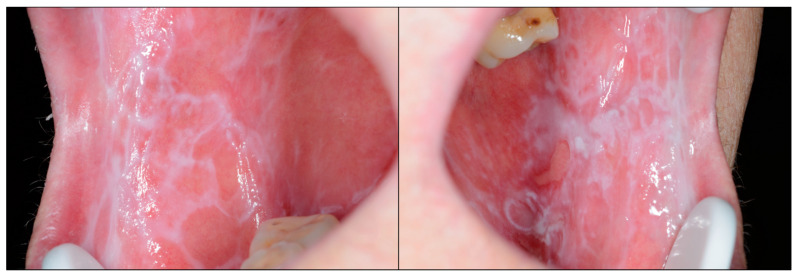
Patient presenting bilateral reticular lesions typical of oral lichen planus; in addition, some erosive lesions appear in the left buccal mucosa [4].

In 2020, a group of international oral cancer experts met in Glasgow under the auspices of the WHO Collaborating Centre for Oral Cancer with the aim of reaching and presenting a consensus on a revised classification of oral potentially malignant disorders (OPMDs) recommended nomenclature and definitions for each disorder. As a result of this meeting, a consensus paper was published [3], which also provided the most recent data on the risk of malignancy of these OPMDs. The working group defined OPMD as “any oral mucosal abnormality that is associated with a statistically increased risk of developing oral cancer”. Among these OPMDs, oral lichen planus (OLP) was included. Some of the members of the research group have been invited to participate in this working group (MAGM) or are authors of some research that has supported the consensus paper (PRG), collaborating essentially as experts in OLP. OLP is defined as a chronic inflammatory disease of unknown etiology and autoimmune nature, mediated by T-lymphocyte aggression directed towards the basal layer of the oral epithelium. OLP presents essentially as white reticular lesions that may or may not be accompanied by atrophic—erythematous—and/or erosive lesions (Figure 1). As mentioned above, OLP is nowadays considered an OPMD. Studies have demonstrated on the basis of evidence that OLP is a frequent disease, affecting 1.01% of the world population and 1.43% of the European population [4]; this prevalence increases significantly and progressively from the age of 40 years [4].

**Figure 2:**
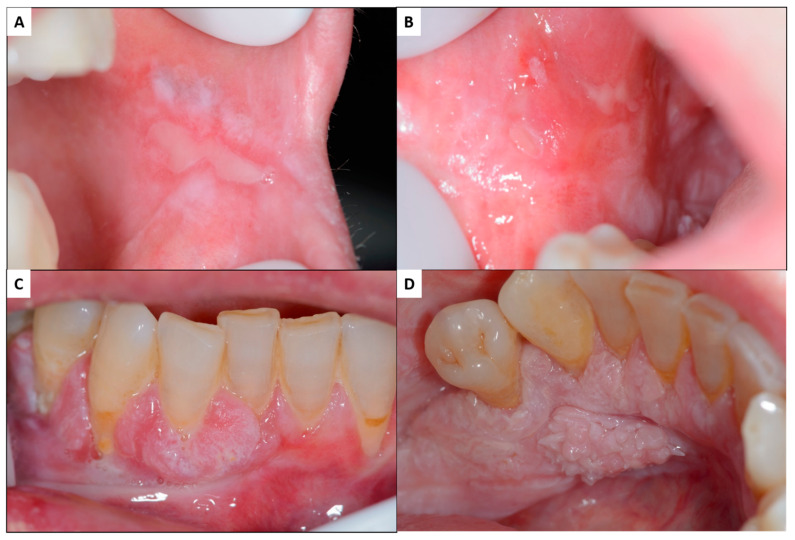
Malignant transformation of oral lichen planus: patient with oral lichen planus affecting both buccal mucosa with erosive atrophic lesions combined with white reticular and plaque lesions (**A**,**B**). In the anteroinferior gingiva, there are exophytic tissue masses, both in the vestibular and lingual areas, which, in the histopathologic, study proved to be oral squamous cell carcinomas (**C,D**) [5].

In the same way, it has also been demonstrated on the basis of evidence that the malignancy rate of the disease constitutes 1.14% of cases [5], although it has been reported that one of the factors that most affects the risk of malignancy published for this disease is the methodological quality of the studies on the subject [6], such that the studies with the highest methodological quality presented a risk of OLP malignancy of 2.28%. It has been published that the risk of OLP progression to cancer (Figure 2) has been systematically underestimated [7,8] and have proposed a series of criteria that should be followed in OLP malignancy studies to improve their design and methodological quality [6,9].

The molecular mechanisms involved in the malignant transformation of OLP are largely unknown, although they seem to be related, as in other autoimmune diseases, to the oncogenic effects linked to the chronic inflammatory aggression established in this lesion [8,10]. Our group has developed in recent years a line of research that has resulted in the publication of several primary-level studies [11,12,13,14,15,16,17,18,19]. In general terms, the malignant transformation of OLP probably is due to the molecular alterations that the inflammatory infiltrate generates in the basal cell layer of the attacked oral epithelium and to the effects induced by the chemokines and growth factors produced by the inflammatory infiltrate on the basal cells, which in turn respond with an increase in their proliferative activity and an inhibition of the apoptotic mechanisms. All of this generates a favorable background for the development of cancer-predisposing mutations. In the same way, the failure in the activation of the main tumor suppressor genes (p53) [18] may constitute a final key mechanism in the malignant transformation of this predisposed field. Another relevant conclusion has been that, since the molecular alterations discussed extend widely along the oral mucosa, the risk of a final molecular event concluding in the appearance of a malignant clone extends also to the overall oral mucosal surface affected by OLP, which would explain the high percentage of malignant OLP cases developing multiple oral carcinomas [20] and provide a molecular justification for the current consideration of OLP as a malignant field [21,22].

However, some studies interested in this topic [23,24,25,26,27,28,29,30,31,32,33,34,35] constitute primary-level studies that consequently provide a limited level of evidence. This is the reason for this scoping review of systematic reviews and meta-analyses that aims to present the current knowledge on the expression of the cancer hallmarks [2] in OLP based on the evidence, with the objective of pointing out evidence gaps [36,37], proposing new lines of research, and achieving a better understanding of the molecular mechanisms involved in OLP and its malignization, which will allow the design of strategies applicable to its prevention.

## 2. Materials and Methods

### 2.1. Search Strategy

This scoping review adhered to the Preferred Reporting Items for Systematic reviews and Meta-Analyses extension for Scoping Reviews (PRISMA-ScR) [38]. Cochrane Database of Systematic Reviews (aka Cochrane Library), DARE Database of Abstracts of Reviews of Effects, MEDLINE (via PubMed) and Embase databases were searched for systematic reviews published before our upper search limit date (October 2022), with no lower date limit. A comprehensive search (Supplementary Materials) was designed considering the PRESS initiative [39], conducted by combining thesaurus terms used by the databases (i.e., MeSH and EMTREE) with free terms and built to maximize sensitivity. The keywords were “oral lichen planus” jointly with hallmarks of cancer, biomarkers and oncogenic-related processes [2]. Based on the function of these genes/proteins, this research catalogued them to fit the hallmarks of cancer. Synonyms terms of the proteins and genes included in the original Hanahan and Weinberg paper, as well as proteins and genes with equivalent functions applicable to a particular hallmark, were also checked through the HUGO Gene Nomenclature Committee, https://www.genenames.org). Keywords were combined jointly with an optimal search filter specifically designed by the Centre for Reviews and Dissemination (CRD) for retrieving systematic reviews and meta-analyses [40,41]. An additional final screening was performed handsearching the reference lists of retrieved included studies and using Google Scholar. All references were managed using Mendeley v.1.19.8 (Elsevier. Amsterdam, The Netherlands); duplicate references were eliminated.

### 2.2. Eligibility Criteria

Systematic reviews, with or without meta-analysis were included, evaluating the hallmarks of cancer, biomarkers and/or related oncogenic processes [2], in the context of oral lichen planus. A “systematic review” was defined as a review clearly formulating a research question and using systematic and explicit methods (minimally a search strategy and eligibility criteria) to identify, select and critically appraise relevant research and to collect and analyze data from the studies that are included in the review [42,43]. No restrictions were applied in relation to publication language, publication date and characteristics of the primary-level studies included in the systematic reviews (e.g., study design, geographical areas, sex and age of patients, follow-up periods, etc.).

### 2.3. Study Selection Process

Eligibility criteria were independently applied by two authors (MAGM and PRG). Articles were selected in two phases, first screening titles and abstracts for articles apparently meeting inclusion criteria and then reading the full text of selected articles, excluding those that failed to meet the eligibility criteria. Any discrepancies were resolved by consensus. 

### 2.4. Data Extraction

Two authors (MAGM and PRG) extracted data from the selected articles, completing a data collection form in a standardized manner using Excel and Word (v.16/2018, Microsoft. Redmond, WA, USA). Data were gathered on the first author, publication year, study population (i.e., oral lichen planus), sample size (i.e., number of studies), study design (i.e., systematic review with or without meta-analysis), biomarkers and hallmarks of cancer investigated and key results. These datasets were additionally cross-checked in several rounds, solving discrepancies by consensus.

### 2.5. Critical Analysis and Evidence Synthesis

This scoping review was designed and developed closely following the framework of the hallmarks of cancer [2]. This paper examined whether the hallmarks of cancer, biomarkers and oncogenic-related processes have been investigated across systematic reviews in the clinical context of patients with oral lichen planus, in order to explore and synthetize the current knowledge, and to identify potential evidence gaps. The results are shown in descriptive tables and figures, using a systematic methodological approach, and finally, critically discussed in depth.

## 3. Results

### 3.1. Results of the Literature Search

**Figure 3:**
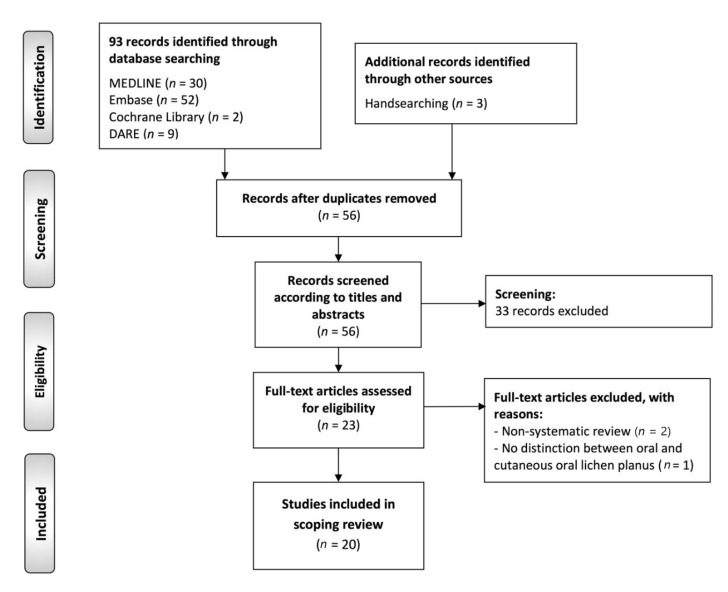
Flow diagram of the identification and selection process of the studies included in this scoping review of systematic reviews.

The flow diagram (Figure 3) depicts the results of the literature search, study identification and selection process. A total of 96 publications were retrieved: 52 from Embase, 30 from MEDLINE (through PubMed), 9 from DARE, 2 from Cochrane Library database of systematic reviews and 3 handsearching reference lists. After duplicate elimination, 56 records were considered potentially eligible and screened according to titles and abstracts, leaving a sample of 23 studies for full-text evaluation. Later, three additional records were full-text excluded, leaving a final sample size of 20 secondary-level systematic reviews meeting all eligibility criteria for critical analysis and evidence synthesis in this scoping review [44,45,46,47,48,49,50,51,52,53,54,55,56,57,58,59,60,61,62,63].

### 3.2. Study Characteristics

Table 1 summarizes the characteristics of the study sample (*n* = 20 studies). The first systematic review identified and included in the scoping review of systematic reviews was published in 2012, and the most recent in 2022. All were designed as secondary-level systematic reviews, and 16 of them performed meta-analysis (80%). The study population was OLP across all studies, as an isolated entity in the most part of them (*n* = 17, 85%), or analyzed jointly with other OPMDs (*n* = 3, 15%).

### 3.3. Critical Analysis and Evidence Synthesis

Table 2 summarizes the evidence derived from the research on hallmarks of oral cancer and the biomarkers investigated in patients with OLP across the secondary-level systematic reviews identified in the scoping review. Table 3 synthetizes the key results categorized according to the classification of the hallmarks of cancer [2]: tumor-promoting inflammation was studied by 17 studies (85%), followed by sustaining proliferative signaling (*n* = 2, 10%) and evading growth suppressors (*n* = 1, 5%). No evidence was found for the rest of the hallmarks of cancer in oral lichen planus (resisting cell death (*n* = 0, 0%); enabling replicative immortality (*n* = 0, 0%); angiogenesis (*n* = 0, 0%); activating invasion and metastasis (*n* = 0, 0%); deregulating cellular energetics (*n* = 0, 0%); avoiding immune destruction (*n* = 0, 0%); genome instability and mutation (*n* = 0, 0%).

## 4. Discussion

### 4.1. Maintenance of Proliferative Signaling

**Figure 4:**
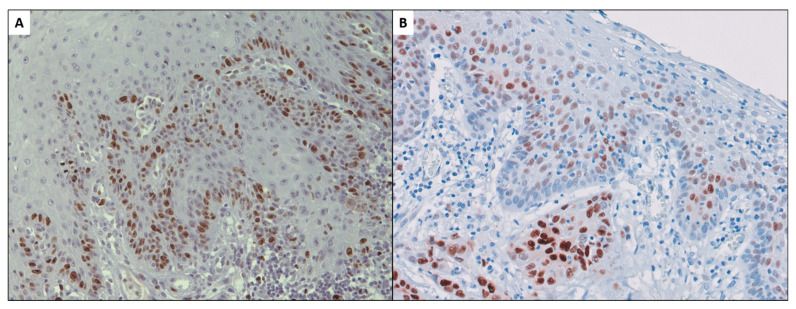
Ki-67 expression in oral squamous cell carcinoma in the earliest stages of invasion (**A**). Cyclin D1 proliferation-stimulating protein expression in early oral carcinoma and adjacent premalignant epithelium (**B**) [71,72].

It is known that probably the most significant defining fact about tumor cells in the oral cavity, and specifically those forming part of oral squamous cell carcinomas (OSCC), is their ability to proliferate beyond the physiological requirements necessary to maintain the structure and function of the normal oral epithelium (Figure 4). The maintenance of an epithelial hyperproliferative phenotype acts as the essential driver for the initiation and progression of oral oncogenesis through the establishment of a state of genomic instability that favors the acquisition of mutations of oncogenes and tumor suppressor genes, indispensable for the clonal expansion of malignant cells and for the acquisition of new cancer hallmarks [1,2,21,22].

**Figure 5:**
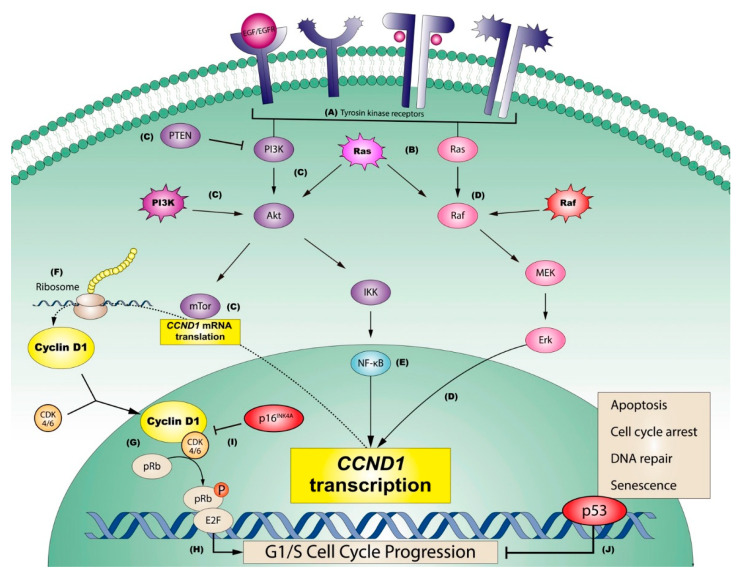
Graphic representation of the most relevant pathways regulating sustaining proliferative signaling in oral squamous cell carcinomas. (**A**) Tyrosine kinase receptors (e.g., EGFR or ErbB2) may be activated on the cell membrane by extracellular growth factors (e.g., EGF) or by constitutive mutations of the genes that encode them. Consequently, (**B**) Ras is downstream activated through the stimulation of these receptors, although Ras can be also activated by mutations, representing an influential central point in oral carcinogenesis, able to stimulate two important downstream oncogenic pathways: PI3K (pathway graphically represented in purple) and/or MAPK (pathway graphically represented in pink). (**C**) The PI3K pathway (PI3K-Akt-mTor, green), which can be blocked by its potent supressor PTEN (**C**), regulates the downstream translation of CCND1 mRNA via mTor (**C**). This pathway can also be constitutively activated by PI3K mutations (**C**). In parallel, (**D**) the endpoint of the MAPK pathway (Raf-MEK-Erk; in pink), which can also be activated by the constitutive mutation of Raf (**D**), is the Erk-mediated transcriptional activation of CCND1 (**D**). A third key pathway in the oncogenic activation of cyclin D1 is (**E**) NF-kB (in blue), which can be activated by IKK, as a consequence of PI3K pathway activation. (**F**) CCND1 transcriptional activation mediated by ERK, and the mTor-mediated translation of its messenger RNA, are both essential for the ribosomal synthesis of cyclin D1, which forms complexes with its binding partners, CDK4/6, that finally translocate to the nucleus. (**G**) Nuclear Cyclin D1-CDK4/6 complexes subsequently activate the retinoblastoma pathway, in which the release of transcription factors E2F is induced by the translocation of a phosphato group, (**H**) with progression from the G1 to the S phase of the cell cycle. The activation of the retinoblastoma pathway can be prevented (**I**) through the potent inhibition of Cyclin D1-CDK4/6 by the product of tumor suppressor gene CDKN2A (i.e., p16INK4), blocking cell cycle advance, or alternatively by the tumor supressor p53 (**J**), which plays an important role arresting cell cycle progression, repairing the damaged DNA or finally promoting apoptosis, in an effort to prevent sustaining proliferation in cancer cells (adapted with permission from [64]).

Among the molecular mechanisms most closely linked to the stimulation of epithelial proliferation are those exerted by the epidermal growth factor receptor (EGFR or ErbB) family and its ligands, the epidermal growth factors (EGF) [64]. The most relevant member of the EGFR family is ErbB2 because it is the most efficient in terms of stimulating proliferation [65,66,67]. EGFR/EGF binding triggers a cascade of downstream hyperproliferative events, among which are the MAPK and PI3K/Akt pathways, which conclude with the activation of genes whose essential mission is to stimulate cell division [68], among which CCND1/Cyclin D1 stands out [69,70,71,72] (Figure 4 and Figure 5). Most EGFRs are overexpressed in human neoplasms [73,74], which essentially occurs by amplification, N- and C-terminal truncation, specific exon deletions, tandem duplications and point mutations [75,76]. These gene alterations lead to the constitutive activation of these receptors without the requirement of binding to their EGF ligands. The evidence of the participation of these pathways in oral carcinogenesis is scarce and is limited to some systematic reviews and meta-analyses [77,78,79], and in OLP, there is no solid evidence (systematic review or meta-analysis) that provides knowledge in this regard, either with regard to EGFR/EGF or CCND1/Cyclin D1. However, there are published primary-level studies—case series—in which the overregulation of these receptors or CCND1/cyclin D1 in OLP has been analyzed [29,80,81,82,83], which could probably allow the performance of a systematic review and meta-analysis providing evidence-based information on this aspect. The activation of the pro-proliferative MAPK and PI3K/Akt pathways can also occur through the stimulation of Ras protein and its downstream targets [64]. The Ras oncogene is frequently mutated in human melanomas [84,85] and is one of the most frequently altered oncogenes in OSCC [86], especially with regard to HRas mutations [87,88], leading to its constitutive activation with hyperproliferation. It should be noted that, despite the importance of this pathway in oral carcinogenesis, there are no primary-level studies and therefore no systematic reviews or meta-analyses that provide information on OLP. The PI3K/AkT pathway includes PTEN as its main inhibitor, which is currently considered a tumor suppressor gene. Alteration with a loss of the function of the tumor suppressor PTEN was the first described mechanism of PI3K/Akt constitutive activation; likewise, this pathway is closely related to mTOR, a serine-threonine kinase that behaves as a target of Akt, which, upon activation, induces the expression of pro-proliferative oncogenes such as MYC and CCND1 [68,89,90]. Evidence-based knowledge of the implications of these proliferative agents (PTEN, mTOR) in oral carcinogenesis is limited to two meta-analyses [77,91], with no information in the form of systematic reviews and meta-analyses in OLP, although some primary-level studies are available [92,93,94].

### 4.2. Evasion of Growth-Suppressive Signals and Development of Resistance to Cell Death

In addition to proliferating, tumor cells should be able to evade the growth suppressor signals that are physiologically established in abnormally proliferating cells; they must also establish mechanisms that make them resistant to cell death, which are distinctive features of cancer [1,2]. These actions are essentially achieved by dysregulating the function of tumor suppressor genes, the best known of which is RB, which encodes the tumor suppressor protein pRb, and the TP53 gene, which encodes p53 (Figure 5).

The tumor suppressor protein pRb acts via cell cycle inhibition in the G1 phase, which, in addition to stopping proliferation, induces cell differentiation and chromosomal stability [95,96]; this constitutes a mechanism by which an essential driver of cancer progression, chromosomal instability, is eliminated. The pRb protein achieves this function by sequestering the E2F transcription factors and thus keeps them away from their target genes. Alterations in the RB tumor suppressor gene are important in tumor initiation and early progression of neoplasms, as has been demonstrated in members of families that inherit alterations in RB alleles that predispose them to the development of familial retinoblastoma [97,98,99], as well as in carcinomas of the uterine cervix and oropharynx, in which HPV viruses, via their E7 oncoprotein, inactivate pRb [100,101]. There is no secondary-level evidence (systematic reviews and meta-analyses) on RB alterations in OLP, and there is only one primary-level study that showed that a loss of RB function was very rare in OLP [102]. This is necessarily a future area of research that should also consider possible oncogenic cooperation in the malignant transformation of OLP between HPV and RB.

Another important tumor suppressor gene is TP53, which encodes the tumor suppressor protein p53. This protein exerts its tumor suppressor functions by inducing apoptosis in cells that suffer irreparable DNA damage or by triggering DNA repair mechanisms in those cases wherein this is possible without compromising the future viability and safety of the cell [103]. The p53 protein also behaves as an inducer of senescence and cell differentiation, which it does through the transcriptional activation of CDKN1A, which transcribes p21, which in turn upregulates the expression of the p16 protein that activates the RB gene. All of this leads to the development of senescence [104]. The aforementioned induction of cell differentiation linked to the actions of p53 makes the cells resistant to reprogramming, thus preventing them from acquiring the characteristics of cancer stem cells [105]; all of this appears to be relevant to the exercise of p53 as a tumor suppressor protein [64]. There is no evidence in the form of systematic reviews and meta-analyses on the implications of p53 for OLP malignancy; neither is there evidence for its subsidiary proteins p21 and p16. A meta-analysis has been published in this regard, showing that alterations in p53 functions behave as significant risk markers for oral leukoplakia malignancy [53], although the evidence in proliferative verrucous leukoplakia and OLP, as a consequence of the lack of primary-level studies developed in case series, has not been reached. This is a necessary line in future research on the subject.

In addition to the above, a powerful source of growth-suppressive signals, both in vitro and in vivo, comes from the maintenance of close cell–cell contact, a phenomenon known as “contact inhibition”. The loss of contact inhibition operates in human carcinogenesis, and some of the mechanisms that regulate it have recently been elucidated. In this regard, Merlin, a tumor suppressor protein encoded by the NF2 gene, has been shown to be altered in neurofibromatosis type 2, an inherited disease that predisposes the bearer to the development of tumors, essentially schwannomas [106,107,108]. The tumor suppressor effects of Merlin are exerted via the promotion of contact inhibition, which it does by facilitating the coupling of the adhesion molecule E-cadherin to EGFR receptors; thus, in addition to maintaining cell–cell junctions, it competes with EGFs for EGFRs, preventing the activation of proliferative pathways linked to these receptors [109,110]. Another mechanism regulating contact inhibition is associated with the actions of the epithelial polarity protein LKB1, which acts as a tumor suppressor and is upregulated in normal epithelium. LKB1 is lost in dissociated epithelia, and this involves the activation of pro-proliferative genes (CCND1/cyclin D1, MYC) [111,112,113,114]. There is no evidence in the form of systematic reviews and meta-analyses on the influence of a loss of contact inhibition on OLP malignancy, nor are there any primary-level studies. This is therefore an absolutely virgin and unexplored line in this field.

The TFG-β chemokine has among its functions tumor suppression [115], which it achieves through the development of cytostatic, differentiation-inducing and proapoptotic effects. TFG-β signaling involves the phosphorylation of its receptor, which in response activates the transcription factor smad that will form complexes with smad4 and stimulate the transcription of its target genes. The tumor-suppressive effects of TFG-β may be lost during oncogenic development for diverse reasons including smad4 mutations or alterations of smad4 antagonists [115,116,117,118,119]. However, as pointed out by Hanahan and Weinberg [2], in tumor cells, TFG-β signaling could also exert perverse functions by inducing in them the mesenchymal epithelial transition phenomenon that endows cells with mesenchymal features, increasing their motility and favoring invasion [120,121]. The aberrant activity of TFG-β could also derive from its ability to produce autocrine mitogens by the tumor cells themselves self-feeding their proliferation [115]. These important and controversial functions have been scarcely studied in OLP case series, including those related to smad4, there being no evidence-based study, so this is an unexplored area.

Other distinctive features of tumor cells are those that point to their ability to resist cell death. The main mechanism of cell death is apoptosis or programmed cell death that develops in those cells with severe and irreparable DNA lesions. This process, in addition to preventing the clonal expansion of cells with highly altered DNA—a highly oncogenic situation—allows these cells to be eliminated without spilling their contents into the extracellular medium and therefore without generating inflammation [122,123,124,125]. The development of apoptosis is established by two different pathways: the extrinsic or death signal receptor pathway involves death ligands produced essentially by macrophages and lymphocytes (TNF, FAS-L, TLIA, TRAIL) bound to their receptors (respectively, TNFR, FAS and TRAIL-R1 and -R2, both receptors for TRAIL); This activates procaspase 8, which, with the help of FAS/FADD, activates caspase 3, which is the final executor of apoptosis [122,123,124,125,126,127,128]; the intrinsic pathway of apoptosis is linked to cellular stressful situations, such as hypoxia, cellular toxicity, and exposure to ROS, and it permeabilizes the outer mitochondrial membrane, releases cytochrome c into the cytoplasm and activates procaspase 9 and finally caspase 3, which, here too, is the executor of cell death. Some apoptosis-activating proteins, such as Bax and Bak, necessary for the permeabilization of the outer mitochondrial membrane, are regulated by anti-apoptotic proteins, whose main exponent is Bcl-2, which together with others—Bcl-xL, Bcl-w, Mcl-1 and A1—bind and inactivate Bax and Bak [124]. Tumor development obviously requires overcoming apoptotic mechanisms [123,124]. Tumor cells can overcome apoptosis by various pathways, although TP53 mutations, the overregulation of Bcl-2 and Bcl-xL, the underregulation of Bax and other proapoptotic [64] mechanisms are particularly noteworthy.

Autophagy is an additional response mechanism of a cell to stressful situations, mainly used in the event of nutrient deficiency [129]. The cell thus breaks down to offer the resulting catabolites for the purpose of biosynthesis and energy production. This process is regulated by different pathways, although the function of beclin-1, a protein of the BH3-only family, linked to Bcl-2/Bcl-xL, seems to be essential in this process, which to develop autophagy requires release from this binding. Autophagy is currently considered a tumor-suppressor mechanism [130], although it could also be considered an adaptive mechanism of tumor cells to survive in the hypoxic environment in which they frequently develop [126,129], an example of the capacity of neoplasms to take advantage of some physiological functions of normal cells for their own benefit.

Necrosis is a type of cell death that eliminates detritus in the environment generating an inflammatory process [131,132,133]. The inflammatory stimulus thus mediated aims to eliminate necrotic debris. There is now evidence to support the benefit associated with inflammation that tumor cells receive [133]. It could thus be that necrosis is not a random process, strictly speaking secondary to stressful cellular situations, but could be established by tumor cells to benefit from the oncogenesis-promoting actions associated with inflammation [131,134].

There is no evidence on the influence of apoptosis, autophagy or necrosis and their dysregulation in OLP malignant transformation, which is especially surprising because some of these processes involve the inflammatory infiltrate, a fact so relevant in the development of OLP. However, results do appear in case series on the expression of some pro- and anti-apoptotic molecules in OLP [12,13,135,136,137,138,139,140] that could serve to construct meta-analyses in the future.

### 4.3. Enabling Replicative Immortality

Human cells physiologically present a limited survival program—linked to a predetermined number of proliferative cycles—that aims to eliminate aging cells that will be replaced by progeny better equipped to fulfill the functions for which they were created, thus minimizing the risks of aging [64]. The main mechanism involved in this process is mediated by the shortening of telomeres, which are non-coding fragments of DNA consisting of hexanucleotide tandem repeats (TTAGGG), located at the ends of chromosomes, whose mission is to protect DNA [141]. Each proliferative cycle shortens the telomeres to the point of severe telomere deterioration, which finally unprotects the DNA and triggers, in extremis, a cellular phenomenon called crisis involving cell death [142]. Extreme telomere shortening is linked to the appearance of chromosomal aberrations that activate TP53 and generate apoptosis [143,144]. In view of this, the great majority of malignant cells will be eliminated, by virtue of TP53 activation, among other mechanisms, although some of them, if tumor-suppression pathways fail, will manage to establish initial clones that will begin their expansion. For the survival and expansion of these initial clones, it also seems necessary that the incipient tumor cells acquire prolonged survival by avoiding the extreme shortening of their telomeres [2]. This is achieved through the mediation of a DNA polymerase enzyme, telomerase, which binds hexanucleotide fragments to telomeres. It is now known that telomerase is expressed in most tumor cells, including oral cancer [145,146,147,148,149,150,151,152,153,154,155,156,157,158,159,160,161,162,163,164,165], conferring resistance to apoptosis and survival. Some evidence in breast cancer has reported that tumor cells may be able to late-activate telomerase for their own benefit. By this mechanism, reduced telomerase activity in the early stages of malignant transformation, in which only the functions of tumor-suppressor genes would fail, would facilitate by telomere shortening the acquisition of cancer-linked nonclonal chromosomal aberrations; later, telomerase activation would promote the survival and expansion of fully established malignant clones [166,167]. It should be pointed out that this important mechanism linked to malignant transformation and progression has been very little studied based on the evidence in oral cancer [168], there being no information (systematic reviews, meta-analyses or primary-level studies) regarding OLP malignization.

### 4.4. Induction of Angiogenesis

Human tumors must develop a vasculature network that allows them to nourish themselves and eliminate catabolites derived from their metabolism, which is essential for tumor progression. This process, called neoangiogenesis, is established in very early stages of malignant transformation, even in preinvasive phases [169]. The ability to generate new blood vessels is nowadays an indisputable characteristic of tumor cells [2].

The process of neoangiogenesis is regulated by interactions of positive and negative stimuli. The main proangiogenic actions are derived from the vascular endothelial growth factor (VEGF) family, of which VEGF-A is the most representative member. These factors are ligands and activators of tyrosine kinase receptors (VEGFRs) [64]. VEGF production can be autocrine -by endothelial cells- or paracrine -production derived from tumor or stromal cells-. Interestingly, paracrine production of VEGF can come from peritumoral inflammatory cells or from cells that integrate the inflammatory infiltrate that frequently accompanies premalignant processes in different locations, including the oral cavity (macrophages, neutrophils, mast cells) [170,171]. It is very interesting to note that, in OLP, there are no secondary-level studies -of high evidence- on the development of neoangiogenesis and its influence on the malignization of the disease. Likewise, there is very little information derived from primary-level studies -case series- on the regulation of proangiogenic factors in OLP. This is surprising, considering that the paracrine production of proangiogenic factors derived from inflammatory infiltrate cells should hypothetically occur in OLP. This unexplored line of research should be addressed in the future.

### 4.5. Activation of Invasion and Metastasis

Unquestionably, a distinctive feature of tumor cells is their ability to metastasize, extending the neoplastic process to distant organs or regional lymph nodes [2,64]. The development of metastasis can be a very early phenomenon in carcinogenesis that can even be gestated in preinvasive phases [172]. The metastatic process must follow a series of orderly phases that include invasion into tissues peripheral to the tissue of origin, intravasation, transit through the blood and lymphatic system, extravasation, establishment in distant tissue in the form of micrometastases and, finally, the development of macrometastases [173].

The invasive phase is mediated by a cellular program called epithelial mesenchymal transition (EMT) [174,175,176,177], whereby the epithelial cells lose their polygonal morphology and acquire a mesenchymal spindle-shaped appearance. The most relevant molecular fact in EMT is the loss of the expression of the adhesion molecule E-cadherin [178], which favors the cells to detach from each other and acquire motility. In addition, these cells in EMT may begin to express vimentin -a typical mesenchymal marker- [179]. A relevant fact of the EMT process is its reversible character, so that these cells can re-express E-cadherin and recover both their inter-cellular junctions and their polygonal epithelial morphology, which seems necessary for metastatic colonies to successfully implant and reach the category of macrometastasis [179]. The EMT phenomenon is governed by the actions of some transcription factors (Snal, Slug, Twist, zab1/2) that activate genes involved in the process [180,181,182,183,184], especially in the loss of E-cadherin [185,186,187]. An interesting observation refers to the fact that the EMT phenomenon is not homogeneous in a tumor, appearing more clearly in the peripheral areas, just in the invasion zones, which suggests that perhaps some stromal stimuli or a factor from the peritumoral inflammatory infiltrate may constitute driving forces of EMT [188,189]. This is of interest in OLP, wherein the influences of the inflammatory infiltrate are evident and wherein can be observed an alteration of the morphology of the basal layer of the epithelium—liquefactive or vacuolizing degeneration—that has been systematically related to the autoimmune aggression to which these cells are subjected but which could also, at least hypothetically, be partly due to an EMT phenomenon. There is no evidence (systematic reviews and meta-analyses) on EMT in OLP, nor on the molecular events that occur in it (loss of E-cadherin, vimentin expression, expression of the transcription factors mentioned); on the other hand, there are very few primary-level studies on these aspects, so that their number would not allow the performance of meta-analyses with solvency. Therefore, it is advisable to begin research in this field.

### 4.6. Enabling Characteristics

Hanahan and Weinberg [2] pointed out in their studies that the distinctive characteristics of cancer were achieved by neoplastic cells in a progressive manner thanks to the existence of a series of conditions that made their acquisition possible; among them the authors pointed out the instability of the genome of premalignant and malignant cells and the existence of an inflammatory state underlying most preinvasive lesions, or developed around tumor growths, which exerts oncogenic effects.

The concept of genomic instability refers to the predisposition of the genome of tumor cells to develop mutations and other chromosomal aberrations during cell division and, as it is logical to assume, this is greater as cell proliferative activity increases. Therefore, the hyperproliferative state observed in many malignant cells, and also in premalignant lesions, is the essential reason for genomic instability and the driving force for the acquisition of cancer hallmarks, which, once established, will be clonally transmitted to neoplastic cell progenies [64]. Genomic instability develops secondary to the failure of tumor-suppressor mechanisms essentially, but not exclusively, mediated by alterations in the TP53 gene [190]. Another group of genes called caretaker genes (e.g., BRCA 1 and 2) have also been shown to cause genomic instability in some oncogenic models, such as breast [188]. Genomic instability also develops secondary to progressive telomere shortening during aging, which deprotects DNA and induces cancer-predisposing chromosome alterations. Telomerase enzyme activity is a preventive mechanism for the acquisition of genomic instability, although, as mentioned above, it could also be used by neoplastic cells, in advanced stages of tumor development, to increase their survival and maintain their clonal expansion. Thus, telomerase activity could also induce, as a paradoxical effect, genomic instability [141]. Likewise, the amplifications of some chromosomal regions, frequent in oral cancer, such as 3p26/28 and 11q13 [21,68,69,128,191,192,193], are linked to the overregulation of some oncogenes that are located in these areas and which, like CCND1, encode oncoproteins cyclin D1) that stimulate proliferation and generate genomic instability.

Another enabling characteristic [2] refers to the chronic inflammation that appears almost constantly in many tumors and in many premalignant lesions in their pre-invasion phase. Although for years, it was believed that the functions related to the peritumoral inflammatory infiltrate were limited to immune defense against the tumor, it is now known that there are remarkable pro-oncogenic actions linked to the inflammatory infiltrate, essentially mediated by chemokines and growth factors produced by inflammatory cells [133,171,194,195]. Indirect evidence attesting to the importance of inflammation in tumor progression comes from the fact that many autoimmune diseases end with tumor development in target organs. This is the case for example with ulcerative colitis, primary biliary cirrhosis, Sjögren’s syndrome and also with OLP, which is indisputably an OPMD [3,5,6,9]. Our group has performed numerous primary-level studies examining the expression of proteins produced by the infiltrate and their effects on cellular kinetics in OLP lesions [12,15,16,17,196], hypothesizing how these influences of autoimmune inflammation may contribute to the malignancy of this disease.

In relation to what it is known about the importance of these enabling characteristics in OLP malignization, the information available to us based on secondary-level studies that provide good scientific evidence is scarce. Systematic reviews and meta-analyses on the mechanisms that condition the development of genomic instability in OLP have not been published. Only one meta-analysis [63] addresses the implications of cyclin D1 oncoprotein overexpression on the risk of the malignization of OPMD. However, this meta-analysis, due to lack of primary-level studies, was unable to provide results stratified by type of oral potentially malignant disorder, including OLP. Regarding the evidence-based knowledge on the actions of the inflammatory infiltrate in the malignization of OLP, as these are mediated by molecules and cells of the lesional microenvironment, they will be addressed in a specific section for this topic.

### 4.7. Reprogramming of Energy Metabolism

The increase in the cellular proliferative state that frequently occurs in cancer cells and in those in the premalignant state implies a series of energetic requirements that the cell must fulfill [2]. In differentiated cells, with little or no proliferative activity, in the presence of oxygen, a type of metabolism called oxidative phosphorylation develops, which converts most of the glucose to ATP. This type of metabolism is highly efficient in terms of energy production [197]. When these cells develop in an oxygen-poor environment, they shift their metabolism to the so-called anaerobic glycolysis with much less energy (ATP) production and more lactate generation. In contrast, proliferative cells, including tumor cells, shift their metabolism toward a type called aerobic glycolysis that produces less ATP but generates components for biomass, such as palmitate [197]. The reason why a proliferative cell changes its metabolism towards aerobic glycolysis, which does not produce too much energy, is probably related to the capacity of this type of metabolic design to produce elements necessary for biomass, since the proliferative state not only requires a certain amount of energy—not excessive—but needs, above all, a large amount of biomass due to the fact that the dividing cells must duplicate all their contents [198]. The first researcher who observed and became interested in this type of tumor cell metabolism in 1924 was Otto Warburg [198,199,200], which is why this type of metabolism has been named after him ever since (Warburg effect).

There is no data on whether this fact develops in oral epithelial cells affected by OLP and whether this has any relation to their ability to become malignant. There are no OLP case series in which these aspects have been investigated and, consequently, no systematic reviews and meta-analyses. However, the subject could be of interest, since in OLP, it has been demonstrated that there is a hyperproliferative state that is probably the substrate of its evolution to cancer and that could, as occurs in other proliferating epithelia, be metabolically sustained by Warburg-type cellular metabolism.

### 4.8. Evading the Antitumor Immune Response

There are a number of observations that together provide evidence that the antitumor immune response is a mechanism linked to tumor elimination and that it significantly improves the prognosis of many neoplasms [201]. It is also assumed that tumor expansion requires the overcoming of these immune-mediated antitumor surveillance mechanisms. This led Hanahan and Weinberg [2] to consider the ability to evade the antitumor immune response as a distinctive feature of cancer cells. Tumors, and also preinvasive–premalignant lesions, are frequently infiltrated by a set of immune cells that are located in different positions in relation to the tumor (close to the invasion front, intratumoral, etc.) or in the proximity of epithelia affected by premalignant lesions. This location of the immune cells, together with their density and functional characteristics, constitutes the so-called “immune context” [202], and their study has shown that not all the cells that make up the immune context of a lesion exert positive functions in terms of containing the advance of the neoplasm. For example, TCD3 class lymphocytes, cytotoxic TCD 8 and TC D45RO cells (memory T cells) generally positively affect the prognosis of patients. Regulatory T cells resulting from the activation of TCD4 cells exert a contradictory role depending on the type of tumor, a negative effect having been found in ovarian cancer probably linked to the ability to block the actions of effector T cells, or a positive prognostic effect in head and neck cancer, perhaps linked to the ability to suppress chronic inflammation and the carcinogenesis-promoting effects linked to it [203,204,205,206,207,208,209,210,211]. In this context, it is possible that they could also exert a preventive effect on the malignant transformation of epithelia subjected to autoimmune aggressions, as is the case with OLP. NK cells have a variable role depending on the phenotype they express. For example, in advanced tumors, they may express an anergic phenotype unable to secrete INF-γ and kill tumor cells, which is probably due to the actions of TGF-β produced by the tumor cells themselves. On the contrary, in early stages of carcinogenesis, the conserved capacity of INF-γ secretion could contribute to the good prognosis of the neoplasm. Interestingly, these actions of NK cells linked to their secretion of INF-γ could decrease the likelihood of a premalignant epithelium, such as OLP, concluding in the development of a fully established neoplasm. The role of B cells may be relevant in promoting the early stages of oncogenesis through their ability to produce IL-10 and IgG that activate the M2 pro-tumor phenotype of macrophages [212]. Chemokines and cytokines may also play roles in the antitumor response, essentially CX3CL1, CXCL9 and CXCL10 [213], although other types, such as the chemokine RANTES, have been reported as a possible promoter of OLP malignancy [8,14,15,16,196]. Finally, numerous papers [214,215,216] report that the PD-L1 protein overexpressed in the membrane of tumor cells can induce apoptosis in T lymphocytes, thus eliminating the antitumor response. This mechanism could operate early in OLP as well [217,218,219,220,221].

Research in relation to OLP is maximal in the field of the characterization of inflammatory cells and cytokines and chemokines, along with their functions. However, all of this will be discussed in the concluding section, wherein a joint view of how all these autoimmune cells and the factors and chemokines they produce can hypothetically generate a microenvironment that is likely to act as a promoter of carcinogenesis will be presented.

### 4.9. Tumor Promotion Linked to Inflammation and Tumor Microenvironment

There is multiple evidence linking chronic inflammation with the development of cancer, such that Hanahan and Weinberg have considered this aspect an enabling characteristic that favors the appearance of neoplasms in different organs and tissues [2]. The sustained inflammatory process increases the risk of developing neoplasms in the bladder, uterine cervix, stomach, intestine, esophagus, ovary, prostate or thyroid [222], and this suggests that chronic inflammatory processes affecting the oral mucosa, as occurs with OLP, could perhaps also favor the development of cancer in this location. Moreover, inflammatory cells and the cytokines are present in almost all tumors and accompany the tissue of premalignant lesions in many organs; also, the dianization of some inflammatory mediators, as well as the use of non-steroidal anti-inflammatory drugs, seems to reduce the risk of cancer and mortality [222].

There are very illustrative examples of how the activation of oncogenes and the inactivation of tumor-suppressor genes can contribute to generating a tumor-promoting microenvironment. This has been clearly demonstrated in medullary thyroid carcinoma, in which a genetic rearrangement affecting the gene encoding the RET protein tyrosine kinase, which appears early and is sufficient and necessary for tumor development, generates a transcriptional program in the tumor cell similar to that which appears in the development of inflammation, with the transcription of growth factors, chemokines and proteases, which favor invasion and metastasis [223,224]. Likewise, the activation of the RAS oncogene and the RAS/RAF pathway also stimulates the production of inflammatory cytokines and chemokines [225,226,227], and activation of the MYC oncogene stimulates the production of IL-1β [228]. The inactivation of tumor-suppressor genes behaves in the same sense as occurs with the von Hippel-Lindau tumor suppressor which, on losing its capacity to degrade HIF-1α (hypoxia-inducible factor 1α), releases all of the functions of this factor, including the induction of TNF-α after its interaction with NFκB [229]; similarly, the suppression of the TGF-β protein (a tumor suppressor) promotes tumor-promoting inflammation essentially by increasing the expression of the chemokines CXCL5 and CXCL12, which attracts myeloid lineage MDSCs whose essential function is to suppress the adaptive immune response to tumors facilitating metastasis [222].

Today it is also known that there are some key points in the connection between inflammation and cancer. NF-κB is a key coordinator of immunity that behaves as a tumor promoter [230] that is activated autocrine in tumor cells as a result of gene alterations including mutations, deletions or amplifications [231]. This transcription factor activates genes encoding the production of inflammatory cytokines and some proteins with known tumor-promoting effects such as COX2 or angiogenic factors; it is also a known promoter of survival via the induction of anti-apoptotic genes (Bcl-2) [232,233,234]. Currently, it is known that NF-κB is involved in the initiation and progression of some types of tumors in tissues where cancer-related chronic inflammation usually occurs, such as the gastrointestinal tract and liver [235,236]; moreover, deficiencies of its inhibitor TIR8 are related to an increased susceptibility to the development of bowel inflammation and carcinogenesis [237,238]. The actions of NF-κB are mediated by IKKB, which phosphorylates the inhibitory complex d NF-κB (IκB), releasing NF-κB, allowing its translocation to the nucleus where it activates its target genes; in this context, it has been observed that the inactivation of genes encoding IKKB keeps NF-κB inactive and eliminates the bowel inflammation and carcinogenesis associated with colitis [235]. STAT-3, another important transcription factor, is a potent inhibitor of apoptosis that endows the cells in which it is activated the ability to evade the immune response [239,240,241].

Tumor-associated macrophages (TAMs), which usually infiltrate tumor tissue or are in the peritumoral microenvironment [242,243], develop the M2 protumor phenotype that promotes tumor growth, tissue remodeling angiogenesis and suppresses adaptive immunity [242,243,244]. IL-10 and TGF-β can promote the M2 phenotype [243,244,245].

Finally, during the process of malignant transformation, premalignant or malignant cells express cytokines or cytokine receptors with oncogenic functions, including the promotion of lymph node metastasis (CXCR4/CXCL12, CXCR-1, -2, -3, -5, -7, CX3CR1, CCR1, CCR7, CCR9 and CCR10) [246,247,248,249,250,251,252,253,254,255]. Among all of them, the tumor-promoting functions related to the actions of TNF-α [256], an important promoter of the mesenchymal epithelial transition phenomenon [257], should be highlighted.

Information on this enabling condition associated with the promotion of oncogenesis linked to the inflammatory infiltrate and the effects of chemokines released into the microenvironment has received the most attention in OLP, which seems logical considering that OLP is essentially a disease mediated by chronic inflammatory aggression. Eight published meta-analyses on interleukin production in OLP patients indicate that interleukins have mostly been investigated by ELISA or other immunoassay techniques, measuring their expression levels in saliva or serum/plasma. All of the interleukins investigated have shown higher levels in OLP samples vs. healthy controls. In this regard two meta-analyses have published data for IL-4 [48,50] and two for IL-6 [58,59], and in single meta-analyses, separate data have been published for IL-8 [61] and IL-17 [54]. Finally, two different meta-analyses have investigated polymorphisms showing that the 592C/A polymorphism of IL-10 is significantly more frequent in OLP patients than in healthy controls, in contrast to the other polymorphisms investigated, which did not show significant differences (IL6-174G/C, IL10-819C/T and IL10-1082G/A) [55,56]. Two meta-analyses have published data on TNF-α [44,57]. One of them [57] showed that salivary TNF-α expression levels were significantly higher in OLP patients compared to healthy controls, while another meta-analysis [44] has reported that TNF-α-308G/A polymorphism was not significantly more frequent in OLP patients compared to in the control group. Another meta-analysis [46] has reported that salivary and serum IFN-γ levels were not significantly more frequent in OLP patients than in healthy controls and that this biomarker might not be a risk factor for the development of OLP. It is interesting to note what was reported by a meta-analysis [52] pointing to a significant increase of malondialdehyde, 8-hydroxy-deoxy and nitric oxide in the saliva and serum/plasma samples of OLP patients compared to healthy controls. It should be taken into consideration that malondialdehyde is a carcinogenic metabolite of the COX2 pathway. The same meta-analysis has reported a significant decrease in total antioxidant capacity, uric acid and vitamin C in the saliva and serum/plasma samples of OLP patients compared to healthy controls [52]. This is also interesting because some studies have linked the malignant transformation of OLP to ROS-derived stress. Finally, a systematic review without meta-analysis [45] concluded that serum, salivary and tissue levels of MMP9 are significantly higher in OLP patients compared to healthy controls.

## 5. Conclusions

**Figure 6:**
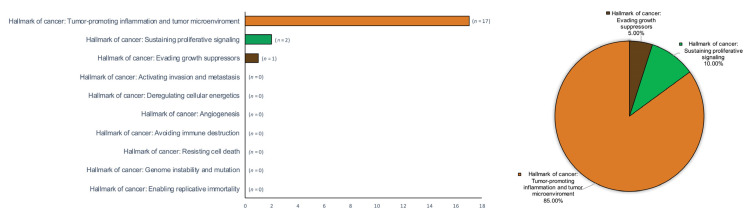
Bar and pie charts graphically summarizing the evidence derived from the research on hallmarks of oral cancer in patients with OLP across secondary-level systematic reviews and meta-analyses. Hallmarks of cancer were ordered by absolute counts (left, bar chart) and relative frequencies by calculating raw proportions, expressed as percentages (right, pie chart).

The current consideration of OLP as an autoimmune disease [8,10] has raised the hypothesis that its malignant transformation could be mediated by the inflammatory infiltrate that systematically appears, in a similar way to what occurs with other autoimmune diseases such as ulcerative colitis or primary biliary cirrhosis. There has been some primary level studies published -retrospective cohorts- on the expression and possible participation of some oncoproteins and tumor-suppressor proteins in the OLP malignization process, together with other papers evaluating cell proliferative activity, as well as the development of apoptosis in OLP epithelial cells [11,12,13,14,15,16,17,18,19,92,93,94,102]. In all of our papers, we have demonstrated the existence of an overexpression of epithelial proliferation-stimulating proteins (cyclin D1 and substance P/NK-1R) in OLP compared to healthy controls [17,19], which has actually translated into a significant hyperproliferative response in this disease, as assessed by Ki-67 expression [14,17,19]. The analysis of the expression of apoptosis markers has shown that the apoptotic phenomenon is unusual in OLP, which we have proven through the study of caspase-3 expression, BAX and also through the use of the TUNEL technique [12,15,16]. This lack of apoptosis is probably, in OLP, due to the active establishment of anti-apoptotic mechanisms mediated by the actions of Bcl-2, which we have found to be overexpressed in this disease [14]. For us, it was inevitable to ask why an epithelium intensely and chronically attacked by an autoimmune inflammatory infiltrate that severely harms the basal epithelial cells, distorting their architecture (vacuolizing degeneration), responds with a proliferative increase and with a marked lack of apoptosis, which would not seem to have any logic. The hypothesis presented derives from the consideration that the most damaging and negative phenomenon for the oral epithelium affected by OLP is the appearance of erosions, which translate into the collapse of an epithelium that has succumbed to the autoimmune aggression, totally losing its regenerative capacity. This epithelial loss—erosions—leaves the more noble structures (blood vessels, nerves, salivary glands…) located in the submucosa, physiologically below the epithelium, unprotected. Thus, this antiapoptotic and hyperproliferative response should aim to prevent epithelial collapse to autoimmune aggression and the appearance of erosions in the OLP. Other evidence derived from the work of our research group in these primary level studies indicates that this response probably derives from the actions of tumor-suppressor proteins, most notably p53, which is overexpressed in OLP [14,18]. We have also shown that this overexpression acts essentially by inducing cell cycle arrest for DNA repair and not by inducing apoptosis [14,18]. Our research group has also pointed out that the overexpression of p53 is essentially due to the normofunctioning wild-type form and not to the mutated form of the p53 protein that has lost its ability to act as a tumor suppressor [18]. The end result would be that an oral epithelium intensely stressed by an autoimmune process that injures epithelial cells essentially responds with increased epithelial proliferation. This will create a landscape in which the development of cancer is a real risk, bearing in mind that in addition some of the chemokines and growth factors in the inflammatory microenvironment may exert proven oncogenic effects. Thus, the risk of developing cancer would be the price that the OLP epithelium must pay in order not to succumb to autoimmune aggression. Thus this risk is probably minimized by the tumor-suppressor actions exerted by p53, which, as we have reported, is frequently overexpressed in OLP. The malignant transformation of OLP-affected epithelium will probably require a failure of p53, probably by mutation. The malignization of OLP is not a common phenomenon, probably as a consequence of the fact that mutations of this gene—TP53—are very rare in OLP [258]. If a mutation with a loss of tumor-suppressor function occurs in a case of OLP, this epithelium would immediately be at high risk of developing cancer, and, since the tissue and molecular phenomena occurring in OLP extend to large areas of the oral mucosa, the risk would not only be limited to the development of one tumor but to the development of multiple carcinomas as an expression of the behavior of OLP as a premalignant field, a fact that has been reliably reported in clinical and meta-analytical studies [20,21]. Our hypothesis therefore appears to answer several of the questions that arise regarding the process of OLP malignization, although we must acknowledge that it has limited experimental and evidence-based support. This paper on cancer-hallmark expression in OLP points out that there are many gaps in knowledge on this aspect. We have pointed out in this scoping review that the hallmarks for which there is most support are tumor-promoting inflammation (17 meta-analyses [44,45,46,47,48,49,50,52,54,55,56,57,58,59,60,61,62]), the existence of sustained proliferative signaling (2 meta-analyses [51,63]) and the evasion of growth-suppressive signals/apoptosis evasion capacity (1 meta-analysis [53]) (Figure 6), and in all of them, the results support our hypothesis. However, it is clear that more primary-level studies and more evidence-based research [259] are required to support the idea that OLP malignization derives from the aggressions of the inflammatory infiltrate and a particular type of epithelial response that, as we have pointed out, aims to preserve the aggressed epithelium.

## Figures and Tables

**Table 1:** Summarized study characteristics.

Total Sample	20 Studies
Year of publication	
Range min (first publication)	2012
Range max	2022
Study design	
Systematic review	4
Systematic review and meta-analysis	16
Study population	
OLP	17
OPMD (including OLP)	3

**Table 2:** Summarized evidence derived from the research on the hallmarks of oral cancer and biomarkers investigated in patients with OLP across secondary-level systematic reviews.

**Hallmark of cancer: Sustaining proliferative signaling**	
Cyclin D1	1 study
Substance P/NK1R	1 study
**Hallmark of cancer: Evading growth suppressors**	
p53	1 studies
**Hallmark of cancer: Resisting cell death**	
no evidences	0 studies
**Hallmark of cancer: Enabling replicative immortality**	
no evidences	0 studies
**Hallmark of cancer: Angiogenesis**	
no evidences	0 studies
**Hallmark of cancer: Activating invasion and metastasis**	
no evidences	0 studies
**Hallmark of cancer: Deregulating cellular energetics**	
no evidences	0 studies
**Hallmark of cancer: Avoiding immune destruction**	
no evidences	0 study
**Hallmark of cancer: Genome instability and mutation**	
no evidences	0 studies
**Hallmark of cancer: Tumor-promoting inflammation and tumor microenviroment**	
Interleukins	8 studies
Oxidative/nitrosative stress and antioxidants	2 studies
TNF-α	2 studies
Cancer stem cells	1 study
Cortisol	1 study
Immunoglobulins	1 study
Interferons	1 study
MMPs	1 study

**Table 3:** Secondary-level systematic reviews and meta-analyses published (*n* = 20).

Systematic Reviews and Meta-Analyses Published in Oral Lichen Planus (OLP)					
Biomarker	Study	Year	Population	Design	Key Results
**Hallmark: Sustaining proliferative signaling**					
Substance P/NK1R	Gonzalez-Moles et al. [51]	2021	OLP	SR + MTA	The Substance P/NK1R complex was expressed by a considerable proportion of patientes with OLP (PP = 61.30%, 95% CI = 51.45 to 70.72), showing a higher diffential expression than benign tumours of the head and neck region.
Cyclin D1	Ramos-Garcia et al. [63]	2019	OPMD (including OLP)	SR + MTA	*CCND1*/cyclin D1 upregulation was significantly associated with a higher malignant transformation risk of OPMD (RR = 2.31, 95% CI = 1.46 to 3.64), including OLP, but specific stratified results could not be estimated.
**Hallmark: Evading growth suppressors**					
p53	Ramos-Garcia et al. [53]	2022	OPMD (including OLP)	SR + MTA	p53 overexpression was significantly associated with a higher malignant transformation risk of OPMD (RR = 1.9, 95% CI = 1.4–2.6), including OLP. The predictive capacity of p53 was especially relevant when assessing the risk of malignancy in oral leukoplakia (RR = 2.22, 95% CI = 1.35 to 3.64), but specific results stratified by OLP could not be estimated.
**Hallmark: Resisting cell death**					
—	—	—	—	—	—
**Hallmark: Enabling replicative immortality**					
—	—	—	—	—	—
**Hallmark: Angiogenesis**					
—	—	—	—	—	—
**Hallmark: Activating invasion and metastasis**					
—	—	—	—	—	—
**Hallmark: Deregulating cellular energetics**					
—	—	—	—	—	—
**Hallmark: Avoiding immune destruction**					
—	—	—	—	—	—
**Hallmark: Genome instability and mutation**					
—	—	—	—	—	—
**Hallmark: Tumor-promoting inflammation and tumour microenviroment**					
Cancer stem cells	Singh Saluja et al. [49]	2019	OLP	SR + MTA	The subgroup meta-analysis for OLP showed that cancer stem cell biomarkers (ALDH1, Bmi1 and CD133, all pooled) were significantly associated with a higher risk of malignant transformation (RR = 6.14; 95% CI = 2.70 to 13.95).
Interleukins	Husein-ElAhmed et al. [54]	2022	OLP	SR + MTA	IL-17 showed significantly higher expression levels in the OLP group assessed by PCR (SMD = 1.35 95% CI = 0.20–2.50), by ELISA (SMD = 2.47, 95% CI = 1.17–3.77) and by flow cytometry (SMD = 3.04, 95% CI = 0.69 to 5.39). Subgroup meta-analyses also showed that the erosive OLPs showed higher IL-17 levels than the reticular form.
	Mehrbani et al. [50]	2020	OLP	SR	Contradictory results were found in the differential expression levels of IL-4 in different OLP samples, and relevant sources of heterogeneity were reported (differences in clinical forms of OLP, research methods, or sample size). It was conlcluded that salivary and serum IL-4 could play a role in oral lichen planus patogenesis.
	Mozaffari et al. [48]	2019	OLP	SR + MTA	Salivary and serum IL-4 levels were higher in patients with OLP compared with healthy controls (MD = 2.67 pg/mL, 95% CI = 2.66 to 2.68, and MD = 6.36 pg/mL, 95% CI = 1.47 to 11.24, respectively), thus indicating that IL-4 may represent a potential biomarker for OLP.
	Mozaffari et al. [59]	2018	OLP	SR + MTA	Salivary and serum IL-6 levels were higher in patients with OLP compared with healthy controls (SMD = 4.53, 95% CI = 1.92 to 7.15, and SMD = 1.48, 95% CI = 0.52 to 2.44, respectively), thus indicating that IL-6 may represent a potential biomarker for OLP.
	Mozaffari et al. [61]	2018	OLP	SR + MTA	Salivary and serum IL-8 levels were higher in patients with OLP compared with healthy controls (MD = 766.32 pg/mL, 95% CI = 394.90 to 1137.75, and MD = 8.38 pg/mL, 95% CI = 3.32 to 13.44, respectively), thus indicating that IL-8 may represent a potential biomarker for OLP.
	Liu et al. [58]	2017	OLP	SR + MTA	Salivary and serum IL-6 levels were higher in patients with OLP compared with healthy controls (SMD = 2.35; 95% CI = 0.50 to 4.19, and and SMD = 2.03; 95% CI = 0.74 to 3.33, respectively), thus indicating that IL-6 may represent a potential biomarker for OLP.
	Shi et al. [56]	2017	OLP	SR + MTA	IL10-592C/A polymorphism was significantly more frequent in OLP than in controls and may be a risk factor for OLP development. But no significant differences were observed for IL6-174G/C, IL10-819C/T, and IL10-1082G/A and OLP susceptibility in any genetic models.
	Qiu et al. [55]	2017	OLP	SR + MTA	IL-10–1082 A/G polymorphism was not significantly more frequent in patients with OLP than in controls and may not be a risk factor for OLP development.
Interferons	Mozaffari et al. [46]	2018	OLP	SR + MTA	Salivary and serum IFN-γ levels were not significantly more frequent in patients with OLP than in healthy controls and may not be a risk factor for OLP development.
TNF-α	Mozaffari et al. [57]	2018	OLP	SR + MTA	Salivary TNF-α levels were significantly higher in patients with OLP compared with healthy controls (MD = 25.90 pg/mL, 95% CI = 15.31 to 36.49), thus indicating that -salivary TNF-α may represent a potential biomarker for OLP. But serum TNF-α levels were not significantly more frequent in OLP than in controls.
	Jin et al. [44]	2012	OLP	SR + MTA	TNF-α-308G/A polymorphism was not significantly more frequent in patients with OLP than in controls and may not be a risk factor for OLP development.
Immunoglobulins	Mozaffari et al. [62]	2018	OLP	SR + MTA	Contradictory results were found for IgA levels, being significantly higher in saliva samples from patients with OLP vs. healthy controls (MD = 71.54 mg/L, 95% CI = 12.01 to 131.07) and significantly lower in serum samples (MD = −0.13 g/L, 95% CI = −0.24 to −0.02), while IgG and IgM levels were not significantly more frequent in OLP than in controls.
Oxidative and nitrosative stress	Wang et al. [52]	2021	OLP	SR + MTA	Significant increases in malondialdehyde, 8-hydroxy-deoxy and nitric oxide were found in the saliva and serum/plasma samples from patients with OLP, in comparison to healthy controls. However, salivary nitrites were not different between the two groups.
	Alamir et al. [47]	2019	OLP	SR	Salivary and serum nitric oxide levels were significantly higher in patients with OLP than in healthy controls, thus indicating that nitric oxide may represent a potential biomarker for OLP.
Antioxidants	Wang et al. [52]	2021	OLP	SR + MTA	Significant decreases in total antioxidant capacity, uric acid and vitamin C were found in the saliva and serum/plasma samples from patients with OLP in comparison to healhty controls. But vitamins A and E, zinc and glutathione peroxidase were not different between the two groups.
Cortisol	Humberto et al. [60]	2018	OLP	SR	Results were controversial across two cross-sectional and seven case-control studies. Salivary cortisol levels were found to be higher in OLP than in healthy controls in 5 out of 9 (55.5%) primary-level studies.
MMPs	Venugopal et al. [45]	2016	OPMD (including OLP)	SR	Serum, salivary and tisular MMP9 levels were significantly higher in OPMD (including patients with OLP) than in healthy controls.

## Supplementary Materials

The following supporting information can be downloaded at: https://www.mdpi.com/article/10.3390/ijms232113099/s1.
