# Supplementary material for: Hallmarks of Cancer Expression in Oral Lichen Planus: A Scoping Review of Systematic Reviews and Meta-Analyses
Source: Int J Mol Sci. 2022 Oct 28;23(21):13099. doi: 10.3390/ijms232113099 (PMC9658487; doi:10.3390/ijms232113099)
Supplement: Supplementary file 1 [file ijms-23-13099-s001.zip › ijms-1999997-supplementary.pdf]

Supplementary Materials

Hallmarks of cancer expression in oral lichen planus: a scoping review of systematic reviews and meta-analyses.

Miguel Ángel González-Moles, Carmen Keim-del Pino, and Pablo Ramos-García

Search strategy

MEDLINE/PubMed (*n* = 30)

("ErbB Receptors"[Mesh] OR "Genes, erbB-1"[Mesh] OR "epidermal growth factor receptor"[all fields] OR "egfr"[all fields] OR erbb*[all fields] OR "EGF Family of Proteins"[Mesh] OR "Epidermal Growth Factor"[Mesh] OR "epidermal growth factor"[all fields] OR "egf"[all fields] OR "Genes, erbB-2"[Mesh] OR "Receptor, ErbB-2"[Mesh] OR "Receptor, ErbB-3"[Mesh] OR "neu"[all fields] OR "erbb2"[all fields] OR "erbb3"[all fields] OR "erbb4"[all fields] OR "cerbb2"[all fields] OR "cerbb3"[all fields] OR "cerbb4"[all fields] OR "her2"[all fields] OR "her3"[all fields] OR "her4"[all fields] OR "cyclin d1"[MeSH] OR ("cyclin"[All Fields] AND "d1"[All Fields]) OR "cyclin d1"[All Fields] OR "cyclind1"[All Fields] OR "ccnd1"[All Fields] OR "ccnd 1"[All Fields] OR "Genes, ras"[Mesh] OR "ras Proteins"[Mesh] OR "ras"[All Fields] OR "hras"[All Fields] OR "kras"[All Fields] OR "nras"[All Fields] OR "Phosphatidylinositol 3-Kinases"[Mesh] OR "pi3k"[All Fields] OR "akt"[All Fields] OR "mtor"[All Fields] OR "pten"[All Fields] OR "NF-kappa B"[Mesh] OR "I-kappa B Kinase"[Mesh] OR "nuclear factor kappa b"[All Fields] OR "nf kappa b"[All Fields] OR "nfkb"[All Fields] OR "i kappa b kinase"[All Fields] OR "ikk"[All Fields] OR "STAT Transcription Factors"[Mesh] OR "Janus Kinases"[Mesh] OR "Signal transducers and activators of transcription"[All Fields] OR "stat"[All Fields] OR "stat3"[All Fields] OR "stat5"[All Fields] OR "janus"[All Fields] OR "jak"[All Fields] OR "jak1"[All Fields] OR "jak2"[All Fields] OR "Mitogen-Activated Protein Kinase Kinases"[Mesh] OR "MAP Kinase Signaling System"[Mesh] OR "Proto-Oncogene Proteins B-raf"[Mesh] OR "mitogen activated protein kinase"[All Fields] OR "mapk"[All Fields] OR "mapkk"[All Fields] OR "mapkkk"[All Fields] OR "braf"[All Fields] OR "mek"[All Fields] OR "erk"[All Fields] OR "Retinoblastoma[Mesh]" OR "retinoblastoma"[All Fields] OR "rb"[All Fields] OR "prb"[All Fields] OR "osrc"[All Fields] OR "pp110"[All Fields] OR "p105-Rb"[All Fields] OR "ppp1r130"[All Fields] OR "p110-rb1"[All Fields] OR "Cyclin-Dependent Kinase Inhibitor p16"[Mesh] OR "p16"[All Fields] OR "cdkn2a"[All Fields] OR "ink4a"[All Fields] OR "Cyclin-Dependent Kinase Inhibitor p15"[Mesh] OR "p15"[All Fields] OR "cdkn2b"[All Fields] OR "ink4b"[All Fields] OR "Cyclin-Dependent Kinase Inhibitor p18"[Mesh] OR "p18"[All Fields] OR "cdkn2c"[All Fields] OR "ink4c"[All Fields] OR "p19"[All Fields] OR "cdkn2d"[All Fields] OR "ink4d"[All Fields] OR "Cyclin-Dependent Kinase Inhibitor p21"[Mesh] OR "p21"[All Fields] OR "cdkn1a"[All Fields] OR "Cyclin-Dependent Kinase Inhibitor p27"[Mesh] OR "p27"[All Fields] OR "cdkn1b"[All Fields] OR "Cyclin-Dependent Kinase Inhibitor p57"[Mesh] OR "p57"[All Fields] OR "cdkn1c"[All Fields] OR "Tumor Suppressor Protein p53"[MeSH] OR " Genes, p53"[MeSH] OR "p53"[All Fields] OR "tp53"[All Fields] OR "Neurofibromatosis 2"[MeSH] OR "Neurofibromin 2"[MeSH] OR "neurofibromatosis 2"[All Fields] OR "nf2"[All Fields] OR "Neurofibromin 2"[All Fields] OR "merlin"[All Fields] OR "lkb1"[All Fields] OR "Transforming Growth Factor beta"[Mesh] OR "transforming growth factor beta 1"[All Fields] OR "transforming growth factor beta 2"[All Fields] OR "transforming growth factor beta 3"[All Fields] OR "transforming growth factor beta"[All Fields] OR "tgfb" OR "tgf beta"[All Fields] OR "Caspases"[Mesh] OR "Caspase 1"[Mesh] OR "Caspase 2"[Mesh] OR "Caspase 3"[Mesh] OR "Caspase 6"[Mesh] OR "Caspase 7"[Mesh] OR "Caspase 8"[Mesh] OR "Caspase 9"[Mesh] OR "Caspase 10"[Mesh] OR "Caspase 12"[Mesh] OR "Caspase 14"[Mesh] OR caspase*[All Fields] OR "Genes, bcl-2"[Mesh] OR "Proto-Oncogene Proteins c-bcl-2"[Mesh] OR "bcl-X Protein"[Mesh] OR "bcl-2 Homologous Antagonist-Killer Protein"[Mesh] OR "bcl2"[All Fields] OR "bax"[All Fields] OR "bclx"[All Fields] OR "bak"[All Fields] OR "bclw"[All Fields] OR "mcl1"[All Fields] OR "Cytochrome c Group"[Mesh] OR "cytochrome c"[All Fields] OR "cyt c"[All Fields] OR "cyc"[All

Fields] OR "NOXA1"[Mesh] OR "NADPH oxidase activator 1"[Mesh] OR "noxa"[All Fields] OR "nadph oxidase activator 1"[All Fields] OR "puma"[All Fields] OR "bbc3"[All Fields] OR "jfy1"[All Fields] OR "bcl2 homology region 3 bh3 only"[All Fields] OR "bh3 only"[All Fields] OR "bim"[All Fields] OR "Autophagy"[Mesh] OR "autophagy"[All Fields] OR "Beclin-1"[Mesh] OR "beclin-1"[All Fields] OR "becn1"[All Fields] OR "atg6"[All Fields] OR "vps30"[All Fields] OR "Necrosis"[Mesh] OR "necrosis"[All Fields] OR "Telomerase"[Mesh] OR "telomerase"[All Fields] OR "tert"[All Fields] OR "Vascular Endothelial Growth Factors"[Mesh] OR ("vascular"[All Fields] AND "endothelial"[All Fields] AND "growth"[All Fields] AND factor*[All Fields]) OR "vegf" [All Fields] OR "Receptors, Vascular Endothelial Growth Factor"[Mesh] OR ("vascular"[All Fields] AND "endothelial"[All Fields] AND "growth"[All Fields] AND factor*[All Fields] AND receptor*[All Fields]) OR "VEGFR"[All Fields] OR "tsp1"[All Fields] OR "tsp"[All Fields] OR "thrombospondin 1"[All Fields] OR "thbs1"[All Fields] OR "thbs"[All Fields] OR "Fibroblast Growth Factors"[Mesh] OR ("fibroblast"[All Fields] AND "growth"[All Fields] AND factor*[All Fields]) OR "fgf" [All Fields] OR "plasmin"[All Fields] OR "angiostatin" [All Fields] OR "Endostatins"[Mesh] OR endostatin*[All Fields] OR "collagen type 18"[All Fields] OR "Pericytes"[Mesh] OR pericyt*[All Fields] OR "rouget cells"[All Fields] OR "Cadherins"[Mesh] OR "e-cadherin"[All Fields] OR cadherin*[All Fields] OR "cd324"[All Fields] OR "cdh1"[All Fields] OR "n-cadherin"[All Fields] OR "CD325"[All Fields] OR "CDH2"[All Fields] OR "Epithelial-Mesenchymal Transition"[Mesh] OR ("epithelial"[All Fields] AND "mesenchymal"[All Fields] AND "transition"[All Fields]) OR "emt"[All Fields] OR snail*[All Fields] OR "slugh2"[All Fields] OR "sna"[All Fields] OR "snah"[All Fields] OR "slug"[All Fields] OR "slugh1"[All Fields] OR "Twist-Related Protein 1"[Mesh] OR "Twist Transcription Factors"[Mesh] OR twist*[all fields] or "bhlh"[all fields] or "scs"[all fields] or "h-twist"[all fields] or "bpes2"[all fields] or "bhlha38"[all fields] or "crs1"[all fields] OR "zeb1"[all fields] OR "zeb2"[all fields] OR "zinc finger E-box binding homeobox 1"[all fields] OR "zinc finger E-box binding homeobox 2"[all fields] OR "tcf8"[all fields] OR "ppcd3"[all fields] OR "bzp"[all fields] OR "zeb"[all fields] OR "areb6"[all fields] OR "nil-2-a"[all fields] OR "zfhep"[all fields] OR "zfhx1a"[all fields] OR "fecd6"[all fields] OR "zfhx1b"[all fields] OR "kiaa0569"[all fields] OR "sip1"[all fields] OR "caretaker"[all fields] OR brca*[all fields] OR "rad51"[all fields] OR "rnf53"[all fields] OR "brcc1"[all fields] OR "ppp1r53"[all fields] OR "fancs"[all fields] OR "fancd1"[all fields] OR "facd"[all fields] OR "fancd"[all fields] OR "rad51a"[all fields] OR "reca"[all fields] OR "hsrad51"[all fields] OR "hst16930"[all fields] OR "brcc5"[all fields] OR "fancr"[all fields] OR "atm"[all fields] OR "ata"[all fields] OR "atdc"[all fields] OR "atc"[all fields] OR "atd"[all fields] OR "tel1"[all fields] OR "telo1"[all fields] OR "DNA Copy Number Variations"[Mesh] OR "Gene Amplification"[Mesh] OR "Sequence Deletion"[Mesh] OR "Glucose Transporter Type 1"[Mesh] OR "glucose transporter type"[All Fields] OR "glut1"[All Fields] OR "hypoxia inducible factor"[All Fields] OR "hif1a"[All Fields] OR "hif-1alpha"[All Fields] OR "pasd8"[All Fields] OR "bHLHe78"[All Fields] OR "HIF2A"[All Fields] OR "HIF-1 alpha-like factor"[All Fields] OR "mop2"[All Fields] OR "pasd2"[All Fields] OR "hlf"[All Fields] OR "bhLhe73"[All Fields] OR "Warburg Effect, Oncologic"[Mesh] OR "warburg"[All Fields] OR "aerobic glycolysis"[All Fields] OR "Isocitrate Dehydrogenase"[Mesh] OR "isocitrate dehydrogenase1"[All Fields] OR "isocitrate dehydrogenase2"[All Fields] OR "idh"[All Fields] OR "idh1"[All Fields] OR "idh2"[All Fields] OR "Tumor Escape"[Mesh] OR (("evading"[All Fields] OR "evasion"[All Fields] OR "escape"[All Fields]) AND "immune"[All Fields]) OR "Neoplastic Stem Cells"[Mesh] OR "cancer stem cells"[All Fields] OR "csc"[All Fields] OR "Endothelial Cells"[Mesh] OR endothel*[All Fields] OR "Angiopoietin-1"[Mesh] OR "ang-1"[All Fields] OR "angiopoietin 1"[All Fields] OR "kiaa0003"[All Fields] OR "angpt1"[All Fields] OR "Receptor, TIE-2"[Mesh] OR "tie2"[All Fields] OR "vmcm"[All Fields] OR "vmcm1"[All Fields] OR "cd202b"[All Fields] OR "tek"[All Fields] OR "angiopoietin-1 receptor"[All Fields] OR "Receptors, Platelet-Derived Growth Factor"[Mesh] OR "pdgf"[All Fields] OR "platelet derived growth factor receptor"[All Fields] OR "Cathepsins"[Mesh] OR cathepsin*[All Fields] OR "heparanase"[All Fields] OR hpse*[All Fields] OR "Myeloid-Derived Suppressor Cells"[Mesh] OR mdsc*[All Fields] OR "Matrix Metalloproteinases"[Mesh] OR "matrix metallopeptidase"[All Fields] OR "mmp"[All Fields] OR "mmt"[All Fields] OR "mmp1"[All Fields] OR "mmp2"[All Fields] OR "mmp3"[All Fields] OR "mmp7"[All Fields] OR "mmp8"[All Fields] OR "mmp9"[All Fields] OR "mmp10"[All Fields] OR "mmp11"[All Fields] OR "mmp12"[All Fields] OR "mmp13"[All Fields] OR "mmp14"[All Fields] OR "mmp15"[All Fields] OR "mmp16"[All Fields] OR "mmp17"[All Fields] OR "mmp18"[All Fields] OR "mmp19"[All Fields] OR "mmp20"[All Fields] OR "mmp21"[All Fields] OR "mmp24"[All Fields] OR "mmp25"[All Fields] OR "mmp26"[All Fields] OR "mmp27"[All Fields] OR "mmp28"[All Fields] OR "mmp23B"[All Fields] OR "Chemokines"[Mesh] OR "ccl"[All Fields] OR "cxcl"[All Fields] OR "Cytokines"[Mesh] OR interferon*[All Fields] OR interleukin*[All Fields] OR lymphokines*[All Fields] OR monokine*[All Fields] OR oncostatin*[All Fields] OR osteopontin*[All Fields] OR "tumor necrosis factor"[All Fields]

OR “tnf-α”[All Fields] OR "macrophages"[Mesh] OR macrophage*[All Fields] OR monocyte*[All Fields] OR histiocyte*[All Fields] OR "Neutrophils"[Mesh] OR neutrophil*[All Fields] OR “polymorphonuclear”[All Fields] OR histiocyte*[All Fields] OR "Cancer-Associated Fibroblasts"[Mesh] OR (“cancer”[All Fields] AND “associated”[All Fields] AND “fibroblasts”[All Fields]) OR “a-sma”[All Fields] OR ("alpha”[All Fields] AND “smooth”[All Fields] AND “muscle”[All Fields] AND “actin”[All Fields]) OR (“stem cells"[All Fields] NOT "cancer stem cells"[All Fields]) OR “hoxa5"[All Fields] OR "homeobox a5"[All Fields] OR "hox1c"[All Fields] OR "hox1"[All Fields] OR "Smad4 Protein"[Mesh] OR “smad4"[All Fields] OR “smad"[All Fields] OR "madh4"[All Fields] OR “mitf"[All Fields] OR “melanocyte inducing transcription factor"[All Fields] OR "ws2a"[All Fields] OR “ws2”[All Fields] OR “bhlhe32”[All Fields] OR "Activating Transcription Factor 2"[Mesh] OR “atf2"[All Fields] OR “activating transcription factor 2"[All Fields] OR "cre-binding protein 1"[All Fields] OR "Retinoic Acid Receptor alpha"[Mesh] OR “rar alpha"[All Fields] OR “rar-a"[All Fields] OR "RUNX1 Translocation Partner 1 Protein"[Mesh] OR runx*[All Fields] OR “aml1-eto"[All Fields] OR “aml1"[All Fields] OR “cbfa2"[All Fields] OR "SOX Transcription Factors"[Mesh] OR "SOXE Transcription Factors"[Mesh] OR “sox10”[All Fields] OR “sry-box transcription factor 10”[All Fields] OR “dom”[All Fields] OR “ws4”[All Fields] OR “ws2e"[All Fields] OR "alpha-ketoglutarate"[All Fields] OR “αkg”[All Fields] OR “a-KG”[All Fields] OR "d-2-hydroxygluterate"[All Fields] OR “d2hg”[All Fields] OR "pancreas associated transcription factor 1a"[All Fields] OR “ptf1a”[All Fields]) AND (“Lichen Planus, Oral”[MeSH] OR “oral lichen planus” [All Fields]) AND ("Meta-Analysis"[pt] OR "meta-analysis"[tiab] OR "Systematic Review"[pt] OR "systematic review"[tiab])

Embase (*n* = 52)

('epidermal growth factor receptor'/exp OR 'erbB-1' OR 'epidermal growth factor receptor' OR 'EGFR' OR 'erbb*' OR 'epidermal growth factor derivative'/exp OR 'epidermal growth factor' OR 'egf' OR 'epidermal growth factor receptor 2'/exp OR 'erbb2' OR 'neu' OR 'epidermal growth factor receptor 3'/exp OR 'erbb3' OR 'erbb4' OR 'cerbb2' OR 'cerbb3' OR 'cerbb4' OR 'her2' OR 'her3' OR 'her4' OR 'cyclin d1'/exp OR 'cyclin d1' OR 'cyclind1' OR 'ccnd1' OR 'ccnd 1' OR 'ras' OR 'hras' OR 'kras' OR 'nras' OR 'Akt signaling'/exp OR 'pi3k' OR 'akt' OR 'mtor' OR 'pten' OR 'NF kB signaling'/exp OR 'nuclear factor kappa b' OR 'nuclear factor kappa b' OR 'nf kappa b' OR 'nfkb' OR 'i kappa b kinase' OR 'ikk' OR 'JAK-STAT signaling'/exp OR 'stat' OR 'Signal transducers and activators of transcription' OR 'stat3' OR 'stat5' OR 'janus' OR 'jak' OR 'jak1' OR 'jak2' OR 'MAPK signaling'/exp OR 'mitogen activated protein kinase' OR 'mapk' OR 'mapkk' OR 'mapkkk' OR 'braf' OR 'mek' OR 'erk' OR 'retinoblastoma'/exp OR 'rb' OR 'prb' OR 'osrc' OR 'pp110' OR 'p105-Rb' OR 'PPP1R130' OR 'p110-RB1' OR 'cyclin dependent kinase inhibitor 2A'/exp OR 'p16' OR 'cdkn2a' OR 'ink4a' OR 'cyclin dependent kinase inhibitor 2B'/exp OR 'p16' OR 'cdkn2b' OR 'ink4b' OR 'cyclin dependent kinase inhibitor 2C'/exp OR 'p18' OR 'cdkn2c' OR 'ink4c' OR 'cyclin dependent kinase inhibitor 2D'/exp OR 'p19' OR 'cdkn2d' OR 'ink4d' OR 'p21' OR 'cdkn1a' OR 'p27' OR 'cdkn1b' OR 'p57' OR 'cdkn1c' OR 'protein p53'/exp OR 'p53 signaling'/exp OR 'p53' OR 'TP53' OR 'neurofibromatosis type 2'/exp OR 'merlin'/exp OR 'neurofibromaatosis 2' OR 'nf2' OR 'neurofibrin 2' OR 'lkb1' OR 'transforming growth factor beta'/exp OR 'transforming growth factor beta 1' OR 'transforming growth factor beta 2' OR 'transforming growth factor beta 3' OR 'transforming growth factor beta 4' OR ' tgfb' OR ' tgf beta' OR 'caspase'/exp OR 'Caspase 1' OR 'Caspase 2' OR 'Caspase 3' OR 'Caspase 6' OR 'Caspase 7' OR 'Caspase 8' OR 'Caspase 9' OR 'Caspase 10' OR 'Caspase 12' OR 'Caspase 14' OR 'caspase*' OR 'bcl-2' OR 'bax' OR 'bak' OR 'bclw' OR 'mcl1' OR 'cytochrome c'/exp OR 'cyt c' OR 'cyc' OR 'noxa1' OR 'NADPH oxidase activator 1' OR 'puma' OR 'jfy1' OR 'bh3' OR 'bim' OR 'autophagy' OR 'beclin-1' OR 'becn1' OR 'atg6' OR 'vps30' OR 'necrosis'/exp OR 'telomerase'/exp OR 'tert' OR 'vasculotropin'/exp OR 'vegf' OR 'vasculotropin receptor'/exp OR 'vegfr' OR 'tsp1' OR 'tsp' OR 'thrombospondin 1' OR 'thbs1' OR 'thbs' OR 'fibroblast growth factor'/exp OR 'fgf' OR 'plasmin' OR 'angiostatin' OR 'endostatin'/exp OR 'collagen type 18' OR 'pericyte'/exp OR 'pericyt*' OR 'rouget cells' OR 'cadherin'/exp OR 'e-cadherin' OR 'cd324' OR 'cdh1' OR 'n-cadherin' OR 'cd325' OR 'cdh2' OR 'epithelial mesenchymal transition'/exp OR 'emt' OR 'snail*' OR 'slug' OR 'slugh1' OR 'slugh2' OR 'sna' OR 'snah' OR 'twist' OR 'twist*' OR 'bhlh' OR 'scs' OR 'h-twist' OR 'bpes2' OR 'bhlha38' OR 'crs1' OR 'zeb1' OR 'zeb2' OR ' zinc finger E-box binding homeobox 1' OR 'zinc finger E-box binding homeobox 2' OR 'tcf8' OR 'ppcd3' OR 'bzp' OR 'zeb' OR 'areb6' OR 'nil-2-a' OR 'zfhep' OR ' zfhx1a' OR 'fecd6' OR 'zfh1b' OR 'kiaa0569' OR 'sip1' OR 'caretaker' OR 'brca*' OR 'rad51' OR 'rnf53' OR 'brcc1' OR 'ppp1r53' OR 'fancs' OR 'fancd1' OR 'facd' OR 'fancd' OR 'rad51a' OR 'reca' OR 'hsrad51' OR 'hst16930' OR 'brcc5' OR 'fancr' OR 'atm' OR 'ata' OR 'atdc' OR 'atc' OR 'atd' OR 'tel1' OR 'telo1' OR 'DNA Copy Number Variations' OR 'gene amplification' OR 'sequence deletion' OR 'glucose

transporter type 1' OR 'glut1' OR 'hypoxia inducible factor' OR 'hif1a' OR 'hif-1alpha' OR 'pasd8' OR 'bhlhe78' OR 'hif2a' OR 'hif-1 alpha-like factor' OR 'mop2' OR 'pasd2' OR 'hlf' OR 'bhlhe73' OR 'warburg' OR 'aerobic glycolysis' OR 'isocitrate deshydrogenase*' OR 'idh' OR 'idh1' OR 'idh2' OR 'tumor escape' OR 'immune' OR 'stem cell*' OR 'csc' OR 'endothel*' OR 'angiopoietin*' OR 'ang-1' OR 'kiaa0003' OR 'angpt1' OR 'tie2' OR 'vmcm*' OR 'cd202b' OR 'tek' OR ' Platelet-Derived Growth Factor' OR 'PDGF*' OR 'cathepsin*' OR 'heparanase' OR 'hpse*' OR 'Myeloid-Derived Suppressor Cells' OR 'mdsc*' OR 'matrix metalloproteinase'/exp OR 'mmp' OR 'matrix metallopeptidase' OR 'mmt' OR 'mmp1' OR 'mmp2' OR 'mmp3' OR 'mmp7' OR 'mmp8' OR 'mmp9' OR 'mmp10' OR 'mmp11' OR 'mmp12' OR 'mmp13' OR 'mmp14' OR 'mmp15' OR 'mmp16' OR 'mmp17' OR 'mmp18' OR 'mmp19' OR 'mmp20' OR 'mmp21' OR 'mmp24' OR 'mmp25' OR 'mmp26' OR 'mmp27' OR 'mmp28' OR 'mmp23b' OR 'chemokine'/exp OR 'ccl' OR 'cxcl' OR 'cytokine'/exp OR 'interferon*' OR 'interleukin*' OR 'lymphokine*' OR 'monokine*' OR 'oncostatin*' OR 'osteopontin*' OR 'tumor necrosis factor' OR 'macrophage*' OR 'monocyte*' OR 'histiocyte' OR 'neutrophil*' OR 'polymorphonuclear' OR 'histiocyte*' OR 'cancer associated fibroblast'/exp OR 'a-sma' OR 'alpha smooth muscle actin' OR 'stem cell*' OR 'hoxa5' OR 'homeobox a5' OR 'hox1c' OR 'hox1' OR 'smad*' OR 'madh4' OR 'mitf' OR 'melanocyte inducing transcription factor' OR 'ws2a' OR 'ws2' OR 'bHLHe32' OR 'Activating Transcription Factor 2' OR 'atf2' OR 'CRE-Binding Protein 1' OR 'Retinoic Acid Receptor alpha' OR 'Rar alpha' OR 'rar-a' OR 'runx*' OR 'AML1-ETO' OR 'AML1' OR 'CBFA2' OR 'sox' OR 'soxe' OR 'sox10' OR 'SRY-box transcription factor 10' OR 'dom' OR 'ws4' OR 'ws2e' OR 'alpha-ketoglutarate' OR 'αKG' OR 'a-KG' OR 'D-2-hydroxygluterate' OR 'd2hg' OR 'pancreas associated transcription factor 1a' OR 'PTF1a') AND ('oral lichen planus'/exp OR 'oral lichen planus') AND ('systematic review':ti,ab OR [systematic review]/lim OR 'meta-analysis':ti,ab OR [meta analysis]/lim)

Cochrane Library (*n* = 2)

MeSH descriptor: [lichen planus, oral] explode all trees

DARE (*n* = 9)

MeSH DESCRIPTOR Lichen Planus, Oral EXPLODE ALL TREES
